# In good company: association between fungal glycans generates molecular complexes with unique functions

**DOI:** 10.3389/fmicb.2012.00249

**Published:** 2012-07-09

**Authors:** Marcio L. Rodrigues, Leonardo Nimrichter

**Affiliations:** ^1^Fundação Oswaldo Cruz – Fiocruz, Centro de Desenvolvimento Tecnológico em SaúdeRio de Janeiro, Brazil; ^2^Instituto de Microbiologia Professor Paulo de Góes, Universidade Federal do Rio de JaneiroRio de Janeiro, Brazil

**Keywords:** chitin, *Cryptococcus neoformans*, glucuronoxylomannan, polysaccharides, glycan association

## Abstract

The biological properties of fungal immunogens have historically utilized testing of isolated molecules. Recent findings, however, indicate that fungal glycans differing in structure and function can interact to form hybrid complexes with unique properties. In the pathogenic yeast *Cryptococcus neoformans*, chitin-like molecules associate with capsular glucuronoxylomannan (GXM) to form functionally distinct glycan complexes. Such interactions between glycans that result in the formation of structures with different functions strongly suggest that additional molecular complexes with unknown properties may exist in fungal pathogens. Moreover, the identification of these novel complexes has stimulated the search of new immunogens with potential to protect human and animal hosts against systemic mycoses.

The surface of fungal cells is rich in polysaccharides and protein- or lipid-bound oligosaccharides (Nimrichter et al., 2005) that are called glycans (Bertozzi and Rabuka, 2009). In the fungal cell wall, polysaccharides, glycoproteins and glycolipids form complex carbohydrate networks that play key physiological functions (Nimrichter et al., 2005), such as providing structural support and regulating extracellular secretion (Rodrigues et al., 2008b; Casadevall et al., 2009). Notably, structural aspects of fungal glycans differ considerably from those found in mammalian cells (Fukazawa et al., 1995; Nimrichter et al., 2005). Therefore, the uniqueness of wall glycans makes these molecules promising targets for antimicrobial drugs, as extensively reviewed in the literature (Fukazawa et al., 1995; Nimrichter et al., 2005; Doering, 2009; Goldman and Vicencio, 2012).

Fungal glycans have diverse effects in the interplay between the fungus and the host (Nimrichter et al., 2005). Carbohydrate-rich molecules can effectively stimulate protective immune defenses (Pirofski, 2001; Casadevall and Pirofski, 2006), but they can also down-regulate host effector responses (Zaragoza et al., 2009). To date, there has been an extraordinarily rich spectrum of fungal glycans identified with activities ranging from activation of innate responses and induction of humoral and cell-mediated functions to inhibiting host effector cell recruitment and dysregulating cytokine responses (Casadevall and Pirofski, 2005, 2006; Lee et al., 2008; Li et al., 2009; Sorgi et al., 2009; Mora-Montes et al., 2011; Vecchiarelli et al., 2011). Examples of fungal glycans showing contrasting biological activities are available in a number of comprehensive reviews and the impact of glycans from the human pathogenic *Cryptococcus neoformans* have especially been investigated (Fukazawa et al., 1995; San-Blas et al., 2000; Pirofski, 2001; Zaragoza et al., 2009; Vecchiarelli et al., 2011).

Experimental models describing structural and functional aspects of fungal glycans have historically used purified molecules, mutants lacking genes coding for glycan-synthesizing enzymes, and specific glycan-binding probes, including antibodies, lectins, and peptides. These classic approaches have traditionally focused on isolated molecules for structural and/or functional testing. Microscopic techniques, however, have clearly revealed a number of molecular associations at the cell surface of fungi (Maxson et al., 2007a,b; Rodrigues et al., 2008a; De Jesus et al., 2009; Fonseca et al., 2009b; Zaragoza et al., 2009; Jesus et al., 2010), which suggests that the study of isolated molecules is insufficient to fully elucidate the functional impact of these complex structures. Inter and intramolecular non-covalent associations keep cell wall structures compacted and prevent extracellular release. These molecular complexes differ in structure and composition from isolated molecules, implying that functional differences may occur. To illustrate this hypothesis, we will focus on *C. neoformans*, in which glycan complexes with unique functions have been recently described.

The surface of *C. neoformans* is mainly composed of glycans that include complex polysaccharides, protein-bound oligomannosides, *N*-acetyl-glucosamine-rich oligosaccharides and glucosylceramides (Rodrigues et al., 2000; Reese and Doering, 2003; Nimrichter et al., 2005; Reese et al., 2007; Zaragoza et al., 2009; Nimrichter and Rodrigues, 2011). The most striking feature of *C. neoformans* is an external glycan capsule, which plays a number of significant functions during infection and is crucial for disease progress (Zaragoza et al., 2009). Classically, the capsule has been defined as a complex surface network composed of mannoproteins and the heteropolysaccharides glucuronoxylomannan (GXM) and glucuronoxylomannogalactan (GXMGal). GXM, the main component of the capsule, is a potent immune modulator that has been suggested as a vaccine candidate (Pirofski, 2001). Interestingly, a monoclonal antibody targeting GXM (Casadevall et al., 1998) has undergone phase I clinical testing for use in the treatment of cryptococcosis (Larsen et al., 2005). GXMGal, a minor capsular component, can induce apoptosis in immune cells (Villena et al., 2008). Cryptococcal mannoproteins are efficient stimulators of T cell-mediated immune responses (Levitz and Specht, 2006). These molecules are stably connected to the cell wall and require γ-radiation or DMSO treatment to be detached from the fungal surface (Maxson et al., 2007a,b).

During the last five years, a number of studies have demonstrated that the complexity of the *C. neoformans* capsule is greater than previously thought (Rodrigues et al., 2009). For instance, GXM can self-aggregate (Nimrichter et al., 2007), producing polysaccharide samples that differ in both biophysical and serological properties from fractions obtained through classical biochemical methods (Frases et al., 2008). In addition, microscopic analyses in combination with gene deletion and biochemical approaches strongly suggest that, within the capsular microenvironment, GXM interacts with other glycans, including α1,3 glucan (Reese and Doering, 2003; Reese et al., 2007), GXMGal (De Jesus et al., 2009), mannoproteins (Jesus et al., 2010), and chitin-like structures (Rodrigues et al., 2008a). These studies have led investigators to question the prior models of the structure of the *C. neoformans* capsule, and have led us and others to ask whether the association of GXM with other glycans produces functionally different molecules. This question has been initially addressed in an experimental model testing the association of GXM with chitin-like structures (Ramos et al., 2012), as detailed below.

Chitin is composed of β1,4 linked units of *N*-acetylglucosamine. This water-insoluble polysaccharide is a scaffold component of the fungal cell wall (Nimrichter et al., 2005), that is not normally accessible to the immune system. During cell division, chitin is hydrolyzed through the activity of chitinases, resulting in the formation of chitooligomers (Kuranda and Robbins, 1991; Adams, 2004). In *Saccharomyces cerevisiae*, these molecules accumulate in bud scars (Powell et al., 2003). However, the distribution of cell wall chitooligomers in *C. neoformans* seems to be unique, as these molecules are intercalated within the capsular network (Figure 1) (Rodrigues et al., 2008a). The wide distribution of GXM in the capsule, in fact, supports the hypothesis that this polysaccharide has the potential to interact with peripheral components, including chitin oligosaccharides.

**Figure 1:**
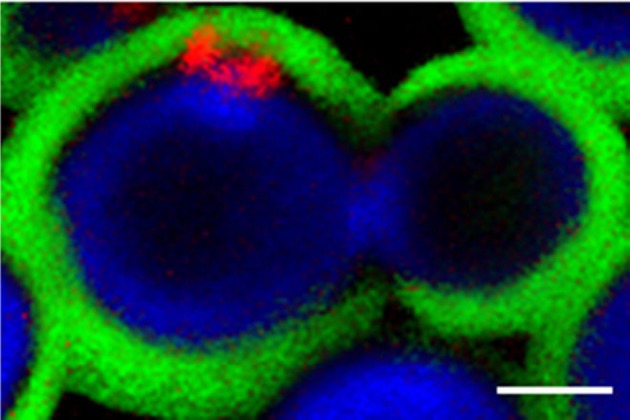
**Confocal section of budding *C. neoformans* cells.** Cell wall chitin (blue fluorescence), capsular GXM (green fluorescence) and chitin oligosaccharides at the cell wall-capsule interface (red fluorescence) were stained as described in Rodrigues et al. (2008a). The image demonstrates that chitin oligosaccharides interact with GXM. Scale bar, 1 μm.

The supposition that GXM and chitin-derived structures interact has been confirmed by a number of approaches (Rodrigues et al., 2008a; Fonseca et al., 2009a,b; Ramos et al., 2012). Using chromatographic and serologic methods in combination with dynamic light scattering, GXM has been shown to interact with chitin and chitooligomers based on the facts that: (1) complexes containing both structures have been isolated from *C. neoformans* cultures, (2) chitooligomers promoted enlargement of GXM fibrils, and (3) exposure of *C. neoformans* cells to an inhibitor of *N*-acetylglucosamine synthesis caused a decrease in capsular dimensions (Fonseca et al., 2009b). Although these studies were in agreement with the ability of *C. neoformans* to form glycan complexes composed of chitin-derived structures and GXM, their production during infection, impact on the host's immune system, and structural determinants regulating this glycan-glycan interaction were unknown until very recently.

A recent study (Ramos et al., 2012) has demonstrated that chitin-GXM association involves non-covalent bonds, large GXM fibers, and depends on the *N*-acetyl amino group of chitin, but not on carboxyl and *O*-acetyl groups of GXM. Importantly, this study shows that glycan complexes formed by GXM and chitin-derived molecules also arise during macrophage infection. Injection of either isolated molecules or the glycan complexes into mice induced distinctly different cytokine responses. In fact, the glycan complexes were efficient in inducing the production of lung IL-10, IL-17, and TNFα, while the cytokine profiles of mice challenged with either GXM or chitin oligomers alone were similar to cytokine levels in control animals. The fact that glycan complex structures produce enhanced immunosuppressive and pro-inflammatory cytokine responses while chitin oligomers and GXM alone did not suggested that cell-associated *C. neoformans* glycans form hybrid structures with unique functions.

The discovery of the formation of functionally distinct glycan complexes raises a number of puzzling questions. For instance, the surface of fungal pathogens is decorated with many different glycans that coexist in several microenvironments (Nimrichter et al., 2005). In fact, many of these molecules are also released into the extracellular space (Rodrigues and Djordjevic, 2012). Therefore, isolated and complexed molecules may interact simultaneously but discordantly with the immune system. Considering the differential response of lung cells to isolated and hybrid molecules in the *C. neoformans* model (Ramos et al., 2012), it is reasonable to postulate that different receptors may be involved in the immune response to each molecular species. The physiologic events regulating the formation of the components of the glycan complex have largely been elusive. For instance, hybrid glycan complexes composed of chitin oligosaccharides and GXM are found in the capsule (Fonseca et al., 2009b) and in the extracellular space (Ramos et al., 2012), implying the requirement of secretory mechanisms for transporting these macromolecules across the fungal cell wall. In fact, GXM is secreted to the cell surface and to the extracellular space by vesicular mechanisms (Yoneda and Doering, 2006; Rodrigues et al., 2007), but secretory processes resulting in the export of chitin oligosaccharides are not known. In this context, it has been established by a number of studies that polymerization and hydrolysis of fungal polysaccharides are surface-associated events (Adams, 2004), with GXM being the only well-known exception (Yoneda and Doering, 2006; Rodrigues et al., 2011). Consequently, it would be reasonable to suppose that the generation of soluble oligosaccharides participating in glycan interactions depends on hydrolytic enzymatic activity.

Chitooligomers are the products of enzymatic hydrolysis of chitin. Chitinase expression is induced during pulmonary cryptococcosis in rodents (Vicencio et al., 2008) and in the bronchoalveolar lavage fluid of asthmatic children (Goldman et al., 2012). The surface distribution of chitooligomers in *C. neoformans* is in fact increased in the lungs of infected rats (Fonseca et al., 2009b). It is also likely that chitooligomers produced through the activity of chitinase are released to the extracellular space, considering their high hydrophilicity and consequent solubility in water. GXM, on the other hand, is constitutively secreted extracellularly (Zaragoza et al., 2009). The concentration of hybrid glycans is severely reduced in cultures with methylxanthine, an inhibitor of fungal chitinases (Ramos et al., 2012). The reduced formation of hybrid glycan complexes as a consequence of chitinase inhibition is in accord with *in vivo* observations demonstrating that chitooligomer detection and capsule enlargement are more evident in host tissues manifesting higher activity of this enzyme (Fonseca et al., 2009b). Therefore, a putative synergistic or additive activity of host and fungal chitinases cannot be discarded.

GXM has the potential to associate with a number of hydrophilic components, mainly because of its high efficiency in the formation of hydrogen bonds. Thus, GXM-chitin interactions probably have other counterparts in *C. neoformans*. Microscopic examinations of *C. neoformans* yeast cells, in fact, support this possibility. Co-staining of cryptococci with antibodies raised to GXM and to α1,3 glucan reveal that α1,3 glucan is widely distributed in the capsule (Cordero et al., 2011). Nevertheless, α1,3 glucan is well known as a cell wall polysaccharide responsible for anchoring *C. neoformans* GXM (Reese and Doering, 2003; Reese et al., 2007), and has not previously been considered as a capsular component. Such unexpected cellular distribution may be linked to enzyme-dependent generation of α1,3 glucan fragments. Glucans are dynamically polymerized and hydrolyzed during cell wall remodeling and yeast replication (Adams, 2004), resulting in the production of soluble glucan oligosaccharides as a natural consequence of cell division. In *C. neoformans*, the presence of the capsule is well-known to slow down the molecular traffic across the cell surface (Nosanchuk et al., 1998; Rodrigues et al., 2000), supporting the possibility that glucan-derived oligosaccharides could be retained within the capsular network after enzymatic hydrolysis. Such mechanism would result in the formation of hybrid microenvironments composed of GXM and glucan-derived oligosaccharides that are compatible with the fluorescence profile observed by Cordero and colleagues (2011) and illustrated in Figure 2. Structural determinations regulating GXM-glucan interactions are still unknown, although hydrogen bonds are likely involved in polysaccharide-polysaccharide interactions (Fonseca et al., 2009b; Ramos et al., 2012). Importantly, these molecules have the potential to form unique glycan complexes, as observed for GXM-chitin oligosaccharides. Such rationale could be also be applicable to other cell wall and capsular components, including β-glucans, GXMGal and mannoproteins.

**Figure 2:**
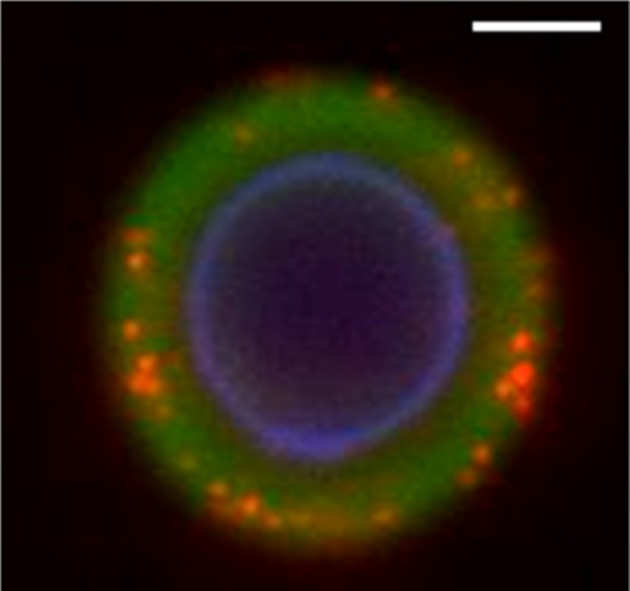
**Fluorescence microscopy of *C. neoformans* after staining for cell wall chitin (blue fluorescence), capsular GXM (green fluorescence) and α1,3 glucan (red fluorescence).** The capsular distribution of α1,3 glucan suggests that the glycan is interacting with GXM and supports the hypothesis that the fungus forms hybrid polysaccharides. For experimental details, see Cordero et al. (2011). Image provided by Dr. Radames, J. B. Cordero. Scale bar, 1 μm.

Surface molecules do not exist in their isolated form in cellular systems and approaches investigating interacting molecules can provide a deeper understanding of complex biological processes than the study of individual purified molecules. The discovery of hybrid glycans with previously unknown functions suggests new venues of investigation on the roles of polysaccharides and glycoconjugates in fungal infections. In addition, the connections between glycan association and functional variation strongly indicate that molecular complexes with still unknown properties may exist in fungal pathogens. This conclusion encourages new perspectives on models aiming at the discovery of protective immunogens.

## Conflict of interest statement

The authors declare that the research was conducted in the absence of any commercial or financial relationships that could be construed as a potential conflict of interest.
